# Pharmacogenomic insights: IL-23R and ATG-10 polymorphisms in Sorafenib response for hepatocellular carcinoma

**DOI:** 10.1007/s10238-025-01576-4

**Published:** 2025-02-08

**Authors:** Asmaa M. El-Sheshtawy, Rehab H. Werida, Monir Hussein Bahgat, Shahira El-Etreby, Noha A. El-Bassiouny

**Affiliations:** 1 Department of Clinical Pharmacy and Pharmacy Practice, Faculty of Pharmacy, Damanhour University, Damanhour, Egypt; 2 Department of Hepatology and Gastroenterology, Mansoura Specialized Medical Hospital, Mansoura, Egypt

**Keywords:** Hepatocellular carcinoma, Sorafenib, Resistance, Pharmacogenomics, IL-23R, ATG-10

## Abstract

Hepatocellular carcinoma (HCC) is the most common primary liver cancer. Sorafenib is the first FDA-approved systemic therapy for advanced HCC. This study investigates the influence of IL-23R (rs7517847) and ATG-10 (rs10514231) genetic polymorphisms on Sorafenib response, survival outcomes, average tolerable dose, and adverse events. This prospective open-label cohort study included 100 HCC patients, assessing IL-23R and ATG-10 genotypes via real-time polymerase chain reaction (RT-PCR). Patient's responses were evaluated using modified RECIST criteria. Statistical analyses evaluated the association of genetic variants with response, progression-free survival (PFS), overall survival (OS), average tolerable Sorafenib dose, and adverse events. IL-23R TT carriers had the highest Sorafenib response rate (80%) compared to GT (13.3%) and GG (6.7%) (*P* = 0.021), while ATG-10 TT carriers had a 13.9-fold increased response likelihood (*P* = 0.001). The T allele in ATG-10 significantly predicted longer PFS (*P* = 0.025) and OS (*P* = 0.011), suggesting a potential prognostic role. IL-23R GG carriers received significantly higher Sorafenib doses than TT (*P* = 0.0174) and GT (*P* = 0.0227), whereas ATG-10 had no effect on dosage. However, its CT genotype was significantly associated with a higher risk of Hand–Foot Syndrome (*P* = 0.012), and independent of dose (*P* = 0.0018). IL-23R and ATG-10 polymorphisms influence Sorafenib response, survival, and tolerability in HCC patients. Genetic screening may improve personalized treatment strategies by optimizing Sorafenib efficacy and minimizing toxicity.

This trial was registered on clinicaltrials.gov with registration number NCT06030895, registered on “September 11th, 2023,” retrospectively.

**Supplementary Information:**

The online version contains supplementary material available at 10.1007/s10238-025-01576-4.

## Introduction

Hepatocellular carcinoma (HCC) is the predominant form of primary liver cancer [1]. The prevalence of HCC has been increasing globally and specifically in Egypt over the past 10 years [2]. HCC is the fourth leading cause of cancer mortality worldwide [3]. The government's targeted screening and follow-up program successfully identified more HCC cases, allowing for earlier diagnosis of patients [4]. Most HCC cases are unresectable when first diagnosed due to structural limitations in the tumor’s location or extensive invasion of the portal or hepatic veins [5]. Limited surgical options for advanced HCC necessitate starting with non-surgical treatment options such as systemic therapy [6]. Sorafenib was the only FDA-approved systemic therapy for a considerable period [7]. In Egypt, Sorafenib is provided by the government for patients with advanced-stage HCC as a part of the government program for hepatic viral disorders [4].

The small drug Sorafenib (Nexavar, Bayer HealthCare Pharmaceuticals) enhances the rate of apoptosis while inhibiting tumor cell growth and tumor angiogenesis [8]. Sorafenib is considered the sole systemic medicine that enhances overall survival in patients with advanced-stage HCC. Compared to a placebo, It demonstrated a median survival improvement of 2.8 months, according to the findings of Sorafenib Hepatocellular Carcinoma Assessment Randomized Protocol (SHARP) trial [8, 9]. Sorafenib was found to inhibit B-Raf, Raf-1, and kinase activity in the Ras/Raf/MEK/ERK signaling pathways, thereby suppressing tumor cell proliferation, and exhibiting anti-angiogenesis and antiproliferation properties [10, 11].

It is known that HCC is one of the most chemotherapy-resistant tumor types [12]. While Sorafenib offers a valuable treatment for advanced HCC patients, numerous individuals fail to benefit from this treatment due to inherent Sorafenib resistance, thereby exposing them to unnecessary harm [6, 9, 10, 13]. The mechanism of Sorafenib resistance is not clear (Kim et al.). This highlights the urgent need for reliable markers to predict Sorafenib response before treatment initiation which can reduce associated side effects and costs. Identification of such biomarkers can also identify patients with a higher likelihood of benefiting from Sorafenib treatment, especially in light of the development of new therapies for HCC [14, 15].

Genes are found to significantly contribute to Sorafenib resistance [16]. Genetic alterations, including genetic variations in angiogenesis-related genes, particularly single-nucleotide polymorphisms (SNPs), have been studied as possible biomarkers for antiangiogenic treatment [13]. Given that SNP evaluation is relatively cheap, can be done at any point during the disease, and is not much impacted by laboratory biases, it would seem to be more favorable as a prognostic marker for Sorafenib responses [12].

A respectable member of the IL-12 cytokine family, interleukin-23 (IL-23) is thought to be a proinflammatory heterodimeric particle. The gene corresponding to the interleukin-23 receptor (IL-23 R) encodes a receptor protein that is present in a variety of immune cells [17]. It is suggested that IL-23R serves a function in different types of cancer expansion and progression [18], including HCC [19, 20]. In the context of HCC, IL-23 is crucial for facilitating cancer growth, progression, and metastasis by reducing CD8+ cell infiltration in tumors and augmenting the immunosuppressive influence of Treg cells. Furthermore, increased IL-23 expression levels in hepatocellular carcinoma (HCC) are associated with advanced TNM staging and metastasis [21]. Furthermore, IL-23R was found to involve in the regulation of innate immunity that influences the progression and severity of HCC [22]. The mechanisms underlying Sorafenib resistance remain incompletely elucidated; nonetheless, immune cells typically play pivotal roles in tumor resistance and have been shown to be significant for the diagnostic and prognostic evaluation of malignancies, potentially correlating with Sorafenib response [23]. And so, IL-23R polymorphisms may also provide an excellent tool to predict whether HCC patients will respond favorably to Sorafenib therapy.

As a part of the Sorafenib mechanism of action, it was found that it promotes autophagy and cell death [24]. Autophagy is a non-apoptotic type II programmed cell death that acts by forming a double-layered membrane-covered spherical organelle called an autophagosome [25]. Several autophagy core genes regulate the formation of autophagosomes and may have a role in the development and spread of cancer, and its response to the therapy [26]. Among these genes, ATG-10 has been demonstrated to play a potentially significant function as a predictor of the development of cirrhosis and HCC with chronic HCV infection [27]. This study aims to ascertain the association between ATG-10 and IL-23R genetic variants that may predict the efficacy of Sorafenib therapy in advanced-stage Egyptian HCC patients. The second aim of this study is to examine the correlation between patient genotypes and overall survival (OS), progression-free survival (PFS), average tolerable dose, and adverse events associated with Sorafenib.

## Material and methods

### Patients

**Fig. 1:**
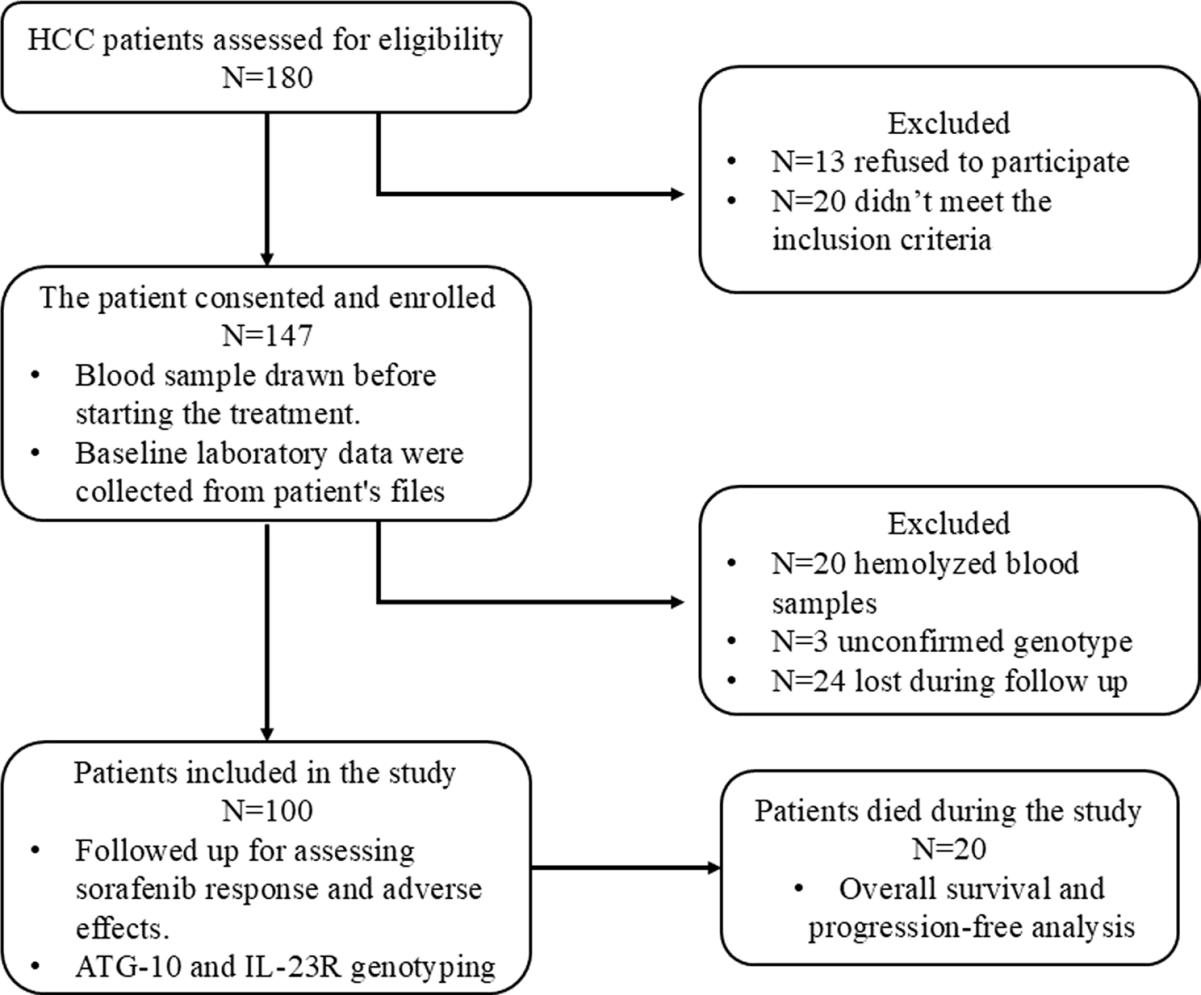
Flowchart of the study

This study was a single-center open-label prospective cohort study, following the STROBE statement for cohort study [28]. Figure 1 summarizes the study flow. There were 180 patients screened between December 2022 and February 2024, 33 patients were excluded, 13 of them declined to participate in the study, and 20 patients did not meet the inclusion criteria. After 152 patients were enrolled, blood samples were taken from them; 20 of them had a low DNA yield due to hemolysis making genotyping impossible for them. The genotype of three cases was not confirmed. Twenty-four patients were lost during the follow-up and their samples were excluded, making a total of 100 patients included in this study. Patients were collected at Specialized Medical Hospital, Mansoura University, El Mansoura, Egypt.

The study included patients diagnosed with HCC, aged ≥ 20 years, with a Performance status of 0–2. The study excluded patients over 75 years old, with renal failure, cardiac disease, HIV infection, pregnancy, Child–Pugh class C, Performance status 3 or 4, or those requiring surgical resection or liver transplant. All enrolled patients were Egyptian. A written informed consent was obtained from participants. The study was conducted according to the guidelines of the Declaration of Helsinki and was approved by the faculty pharmacy Ethics Committee 2022 at Damanhur University (ref no. 1222pp60). This study is also registered on clinicaltrials.gov with No. NCT 06030895, registered on “September 11th, 2023.”

### Blood sampling and laboratory assays

Approximately 3 ml of venous blood was obtained from each patient. The blood was collected into ethylenediaminetetraacetic acid (EDTA) tubes and stored at − 80 °C until DNA was extracted for subsequent genetic analysis and real-time quantitative polymerase chain reaction (PCR), respectively. Other laboratory findings were collected at each follow-up visit, including international normalized ratio (INR), complete blood count (CBC), serum bilirubin, alanine aminotransferase (ALT), aspartate aminotransferase (AST), serum albumin, and serum creatinine.

### Treatment regimen and follow-up

Patients were started at a low dose of Sorafenib 200 mg daily and titrated up to the maximum daily dose of 800 mg if tolerated. Dose modifications were applied when medically indicated. The extent of cirrhosis in liver cancer was assessed using to the Child score classification. Patients were categorized into Child‐Pugh grades A (5–6 points), B (7–9 points), and C (10–15 points). A CT/MRI scan was conducted every 12 weeks for follow-up surveillance. The modified Response Evaluation Criteria in Solid Tumors (mRECIST) was used to assess tumor response to therapy [29]. Treatment with Sorafenib was sustained until disease progression, unacceptable toxicity, or mortality. Continuous safety assessment involved documenting vital signs and clinical laboratory test outcomes, while evaluating the frequency and severity of adverse events in accordance with the National Cancer Institute Common Terminology Criteria for Adverse Events, version 5.0 [30]. Follow-up for side effects was conducted by patient assessments during clinic visits every 4 weeks. Also, telephone interviews were frequently used to check for patients’ safety and adherence in addition to the bill count method.

### IL-23R and ATG-10 genotyping

The sequence of ATG-10 and IL-23R was obtained from the NCBI database. Ensemble browser 90 was used to show all variants to avoid designing primers that overlie SNP sites. We selected a SNP (rs7517847) in the IL-23R gene and a SNP (rs10514231) in the ATG-10 with minor allele frequency > 20%.

DNA was extracted from entire blood samples with a ZYMO DNA extraction kit (Catalog #D4068) adhering to the manufacturer's specifications. The total genomic DNA was quantified utilizing a Nanodrop spectrophotometer (Thermo Fisher Scientific). Furthermore, a gel electrophoresis was performed to ensure the band purity. The samples were preserved at − 20 °C until further examination. Genotyping was carried out using qPCR with TaqMan® allelic discrimination assay software (Applied Biosystems) utilizing Applied Biosystems Step OnePlus 7500 qPCR System. Amplification was performed at 95°C for 10 min, followed by 40 cycles of 95 °C for 15 s and then 60 °C for 1 min and 60 °C for 30 s for annealing and extension. The reading of the two alleles was performed using FAM and VIC filters as instructed by the kit manufacturer.

### Statistical analysis

Data were analyzed using SPSS software (version 26). The Kolmogorov–Smirnov test was used to evaluate the normality of distribution for numerical data. Normally distributed data were reported as mean ± standard deviation (SD) while non-normally distributed data were reported as median and interquartile range. Categorical variables were expressed as numbers and percentages. Differences between groups were compared by independent *t* test or Mann–Whitney U-test. All genetic polymorphisms were examined for deviation by Hardy–Weinberg equilibrium. After 6 months of follow-up, patients were classified into Sorafenib responder and Sorafenib resistance patients. The Chi-square test was performed to identify any significant association between Sorafenib response and genetic polymorphism. Binary logistic regression was used to identify possible factors that may correlate to the drug response. In the statistical analysis, overall survival (OS) and progression-free survival (PFS) were defined as the interval between the date of beginning of Sorafenib treatment to death or last follow-up visit, and to clinical progression or death or last follow-up visit if not progressed. Kaplan–Meier analysis and Cox regression analysis were used for PFS and OS. ANOVA with Tukey’s post hoc test was used to compare average tolerable Sorafenib doses across genotypes. To assess whether genetic variants contributed to adverse events independently of Sorafenib dosage, ANCOVA was performed to adjust for potential confounders. The level of significance is considered at *P* ≤ 0.05. All *P* values represent two-sided statistical tests.

## Results

### Patient characteristics and clinical variables

The study included one hundred HCC patients who were eligible for Sorafenib therapy. Table 1 presents the baseline characteristics of the study participants. The average patient's age at diagnosis was 62 years ± 7.112. Among the 100 patients, 19% were female, and 81% were male. All patients were classified as having advanced stage C according to the Barcelona Clinic Liver Cancer (BCLC) criteria. About 94% of patients tested positive for viral HCV, and 81% were treated with direct-acting antiviral (DAA) treatment. Comorbid diseases such as diabetes and hypertension account for 27% and 29%, respectively. All 100 patients included in the study were diagnosed with cirrhosis and exhibited portal vein invasion, as confirmed by the initial assessment.

**Table 1:** Baseline demographic and clinical characteristics of the study population

	Total 100
	N(%)
Sex (male)	81 (81%)
Age (mean ± SD)	62 ± 7.112
Residency (rural)	88 (88%)
Etiology	
Viral HCV	94 (94%)
Viral HCV + HBV	3 (3%)
Receive DAA	81(81%)
Other disease	
Diabetes	27 (27%)
Hypertension	29 (29%)
Child–Pugh score	
Child–Pugh A	94 (94%)
Child–Pugh B	6 (6%)
ECOG.PS	
ECOG.PS (0)	87 (87%)
ECOG.PS (1)	13 (13%)
Average daily dose of Sorafenib mg	403.4 ± 113.1
Hemoglobin g/dl	12.1 ± 1.84
Serum albumin g/dl	3.78 ± 0.53
INR	1.19 ± 0.17
Serum total bilirubin mg/dl	1.08 ± 0.52
ALT (U/L)	34.75 [21–46.75]
AST (U/L)	44 [27–62]
AFP (ng/ml)	257 [48–1210]
IL-23R, rs7517847	
TT	45 (45%)
GT	45 (45%)
GG	10 (10%)
ATG-10, rs10514231	
CT	51 (51%)
TT	22 (22%)
CC	27 (27%)

HCV, hepatitis C virus; HBV, hepatitis B virus; DAA, direct-acting antiviral; ECOG.PS, Eastern Cooperative Oncology Group Performance scale; INR, international normalized ratio; ALT, alanine aminotransferase; AST, aspartate aminotransferase; and AFP, alpha-fetoprotein

The data were presented as number + frequencies for categorical data, mean ± standard deviation for normally distributed numerical data, and median ± interquartile for non-normally distributed numerical data

### IL-23R and ATG-10 genotypes

The genotype frequencies of IL-23R and ATG-10 are shown in Table 2. Both genes adhered to the Hardy–Weinberg equilibrium. For rs7517847, 45% of patients exhibited homozygosity for the wild-type allele T/T, 45% were heterozygous, and 10% were homozygous G/G. For the second SNP rs10514231, 51% exhibited heterozygous genotypes C/T, whereas 22% of patients were homozygous for the mutant allele T/T.

**Table 2:** Genotype distribution and minor allele frequency among the 100 included patients

SNP	Rs number	Genotype	N 100 (%)	MAF
IL-23R	rs7517847	Homozygote (TT)	45 (45%)	0.33
	T > G	Heterozygote (GT)	45 (45%)	
		Homozygote (GG)	10 (10%)	
ATG-10	rs10514231	Homozygote (CC)	27 (27%)	0.48
	C > T	Heterozygote (CT)	51 (51%)	
		Homozygote (TT)	22 (22%)	

Abbreviation: SNP, single-nucleotide and MAF, minor allele frequency

### Patient’s response to treatment

**Fig. 2:**
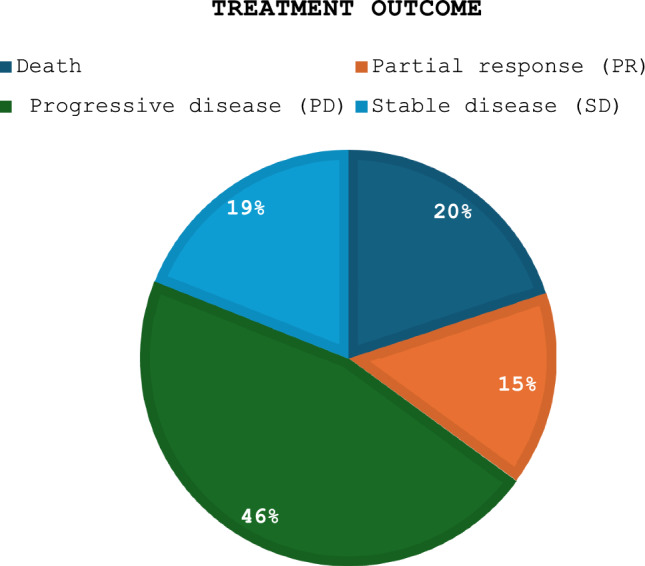
Participant's outcome at the end of the study

Only 80 patients completed the study, as 20 patients died while undergoing treatment with Sorafenib. Among the remaining patients, responses were evaluated using the modified RECIST criteria. Fifteen patients demonstrated a partial response (PR), while 46 exhibited progressive disease (PD) and were transitioned to an alternative treatment. Nineteen patients maintained stable disease (SD), and no complete response (CR) was observed. Figure 2 shows the participant's outcome at the end of the study.

### Association between IL-23R and ATG-10 SNPs and Sorafenib response

The results in Table 3 demonstrate the association between IL-23R and ATG-10 genotypes and the treatment response to Sorafenib in advanced HCC. Among patients with the IL-23R genotype, those with the TT genotype had a higher partial response (PR) rate of 80% (12 out of 16), while the GT and GG genotypes showed lower PR rates of 13.3% and 6.7%, respectively. Stable disease (SD) rates varied significantly across IL-23R genotypes, with TT at 36.8%, GT at 42.1%, and GG at 21.1%. Progressive disease (PD) was most frequent among the TT (45.7%) and GT (50%) genotypes compared to GG (4.3%). The overall response rate (OAR, combining PR and SD) for IL-23R was highest in the TT genotype (55.9%), followed by GT (29.4%) and GG (14.7%), has a *P* value of 0.09, suggesting a trend toward significance.

**Table 3:** Genotype and treatment response to Sorafenib in advanced HCC

Outcome	Total	IL-23R			*P* value	ATG-10			*P* value
	*N* = 80	TT	GT	GG		CC	CT	TT	
PR	16	12	2	1	0.021*	3	3	9	0.003*
		80%	13.3%	6.7%		20%	20%	60%	
SD	19	7	8	4		3	9	7	
		36.8%	42.1%	21.1%		15.8%	47.4%	36.8%	
PD	45	21	23	2		12	29	5	
		45.7%	50%	4.3%		26.1%	63%	10.9%	
OAR (PR + SD)	35	19	10	5	0.09	6	12	16	0.001*
		55.9%	29.4%	14.7%		17.6%	35.3%	47.1%	

CR, complete response; PR, partial response; SD, stable disease; PD, progressive disease; and OAR, overall response

*Significant, *P* value—significance level (alfa = 0.05)

In terms of the ATG-10 genotypes, the TT genotype exhibited the highest PR rate (60%), followed by CC and CT genotypes at 20% each. SD was observed at 15.8% in CC, 47.4% in CT, and 36.8% in TT. PD was most frequent in the CT genotype (52.6%), compared to TT (10.9%) and CC (26.1%). The OAR was highest for the TT genotype at 47.1%, followed by CT at 35.3% and CC at 17.6%, with a statistically significant *P* value of 0.001. These findings suggest that specific IL-23R and ATG-10 genotypes may be associated with differential responses to Sorafenib in patients with advanced HCC, highlighting potential genetic markers for predicting treatment outcomes.

### Logistic regression of Sorafenib response

The association between various clinical factors, genetic polymorphisms, and Sorafenib response in hepatocellular carcinoma (HCC) patients was evaluated using univariate and multivariate logistic regression analyses as demonstrated in Table 4. Among the investigated variables, the ATG-10 rs10514231 TT genotype established a statistically significant association with Sorafenib response in both univariate (OR = 6.4, 95% CI = 1.573–26.034, *P* = 0.010) and multivariate (OR = 9.44, 95% CI = 1.637–54.478, *P* = 0.012) analyses, suggesting a potential predictive role of this genetic variant. In contrast, no significant association was observed for the IL-23R rs7517847 polymorphism in either analysis. Clinical parameters such as age, sex, Child–Turcotte–Pugh (CTP) score, Eastern Cooperative Oncology Group (ECOG) score, Model for End-Stage Liver Disease (MELD) score, and comorbidities including diabetes and hypertension did not show significant correlations with Sorafenib response. However, the presence of metastasis and hepatitis C virus (HCV) infection exhibited trends toward an association, albeit without reaching statistical significance.

**Table 4:** Univariant and multivariant logistic regression analysis between Sorafenib response in HCC patients and ATG-10 rs10514231 and IL-23R rs7517847 genotypes and other factors

Variable	Univariant logistic regression				Multivariant logistic regression			
	Coefficient	Odds ratio	95% CI	*P* value	Coefficient	Odds ratio	95% CI	*P* value
Age	− 0.011	0.989	0.929–1.054	0.736	− 0.007	0.993	0.91–1.09	0.885
Sex	1.178	3.248	0.829–12.718	0.091	0.98	2.669	0.520–13.7	0.240
CTP score	0.226	1.254	0.571–2.754	0.573	− 0.381	0.683	0.198–2.36	0.546
ECOG score	0.346	1.414	0.375–5.333	0.609	0.505	1.657	0.274–10.2	0.583
MELD score	0.164	1.178	0.968–1.433	0.102	0.278	1.321	0.97–1.798	0.077
HCV infection	− 0.327	0.721	0.136–3.813	0.70	1.378	3.966	0.523–30.04	0.182
Metastasis	0.380	1.462	0.60–3.564	0.403	− 0.473	0.623	0.19–2.049	0.436
Diabetes	− 0.418	0.658	0.23–1.881	0.435	− 0.711	0.491	0.08–2.86	0.429
Hypertension	− 0.049	0.952	0.361–2.509	0.921	0.603	1.827	0.416–8.032	0.425
IL-23R rs7517847								
TT (reference)								
GT	Reference		Reference	0.106	Reference		Reference	0.086
GG	− 0.733	0.481	0.183–1.265	0.138	− 0.810	0.445	0.129–1.527	0.198
	1.016	2.763	0.479–15.954	0.256	1.392	4.022	0.545–29.688	0.172
ATG-10 rs10514231								
CC (reference)								
CT	Reference		Reference	0.003*	Reference		Reference	0.004*
TT	− 0.189	0.828	0.252–2.717	0.755	− 0.668	0.513	0.105–2.502	0.409
	1.856	6.4	1.573–26.034	0.010*	2.245	9.44	1.637–54.478	0.012*
Constant	− 0.851	0.427		0.866				

Values in bold with an asterisk (*) represent statistically significant results (*P* ≤ 0.05)

Abbreviation: CTP, Child–Turcotte–Pugh; ECOG, Eastern Cooperative Oncology Group; MELD, model for end-stage liver disease; and HCV, hepatitis C virus

### Association of genetics polymorphism with OS and PFS

**Fig. 3:**
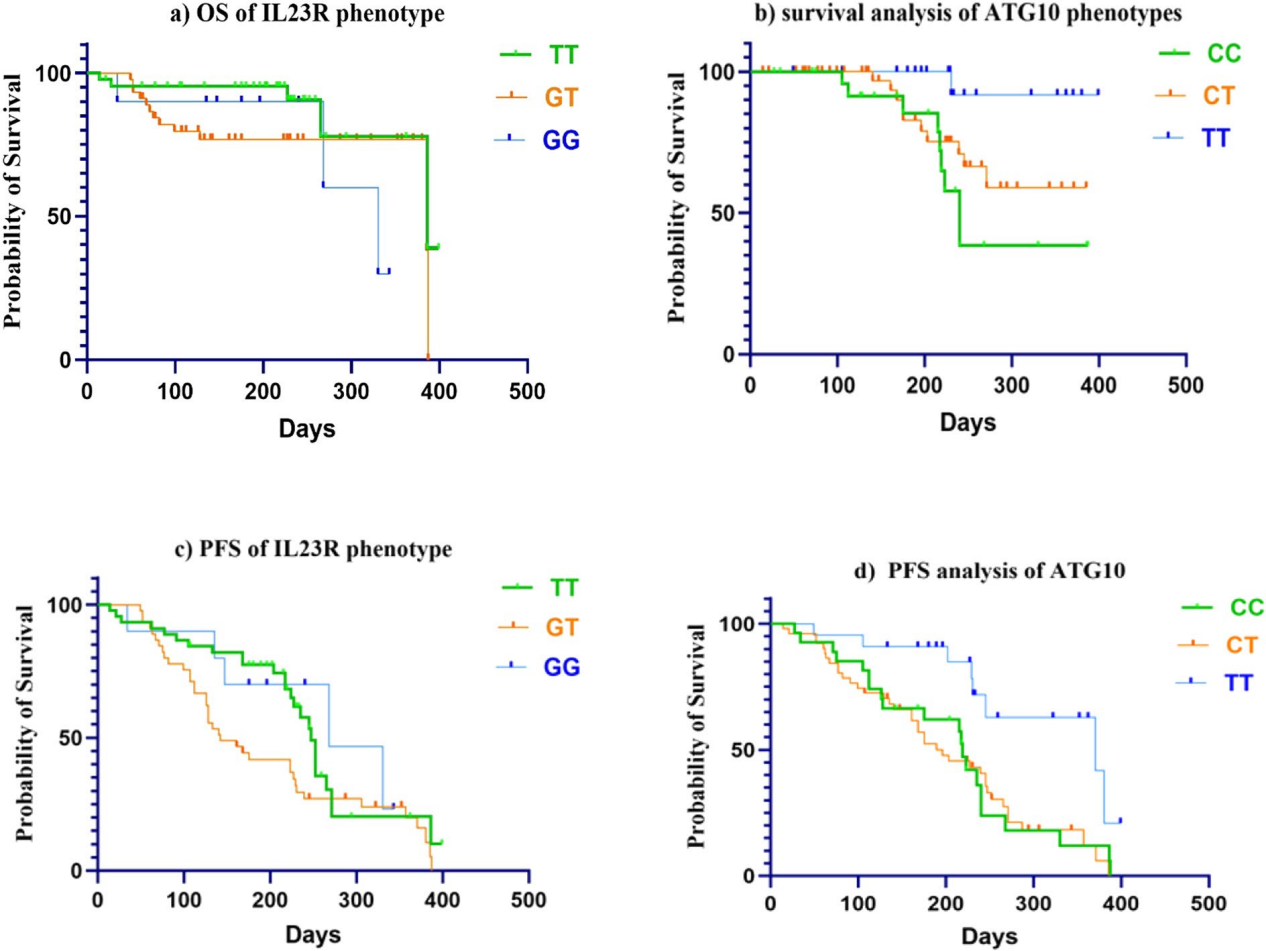
Kaplan–Meier analysis of genotype association with OS and PFS. **a** Kaplan–Meier overall survival (OS) curves of IL-23R phenotype in HCC patients receiving Sorafenib. **b** Kaplan–Meier overall survival (OS) curves of ATG-10 phenotype in HCC patients receiving Sorafenib. **c** Progression-free survival (PFS) curves of IL-23R phenotype in HCC patients receiving Sorafenib. **d** Progression-free survival (PFS) curves of ATG-10 phenotype in HCC patients receiving Sorafenib. The figure was created using GraphPad Prism 10

The impact of genetic polymorphisms on overall survival (OS) and progression-free survival (PFS) in hepatocellular carcinoma (HCC) patients undergoing Sorafenib treatment was evaluated using Kaplan–Meier analysis presented in Table 5 and multivariate Cox regression analysis displayed in Table 6. The IL-23R rs7517847 polymorphism demonstrated a trend toward shorter OS in patients carrying the GT and GG genotypes compared to the TT genotype, although statistical significance was borderline (*P* = 0.076). The mean survival time was 351.3 days (95% CI 311.7–391) for TT carriers, compared to 313.7 days (95% CI 272.5–354.9) for GT and 285 days (95% CI 223.9–347.5) for GG carriers. Furthermore, multivariate Cox regression analysis revealed that the GG genotype was significantly associated with poorer OS (HR = 11.595, 95% CI = 1.664–80.794, *P* = 0.013), suggesting a potential role of this variant in influencing patient survival. The survival curves are illustrated in Fig. 3.

**Table 5:** Association between a genetic variant with OS and PFS in HCC patients

Genes	Genotype	OS			PFS		
		No. event	Mean survival time (days) 95% CI	*P*	No. event	Mean (days) 95% CI	*P*
**IL-23R** rs7517847	TT	5	351.3 (311.7–391)	0.076	25	239.6 (204.6–274.5)	0.408
	*N* = 45						
	GT	12	313.7 (272.5–354.9)		38	195.3 (159.8–230.8)	
	*N* = 45						
	GG	3	285 (223.9–347.5)		5	251.167 (184.3–318)	
	*N* = 10						
**ATG-10** rs10514231	CC	9	289.5 (228.8–350.1)	0.025*	21	207.7 (163.4–252.1)	0.011*
	*N* = 27						
	CT	10	321 (281.9–360.7)		39	198 (166.3–230)	
	*N* = 51						
	TT	1	383 (301.1–354.3)		8	311 (199.4–247.9)	
	*N* = 22						

OS, overall survival; PFS, progression-free survival; and CI, confidence interval

*Significant, *P* value—significance level (alfa = 0.05)

**Table 6:** Multivariate Cox regression analysis for PFS and OS in HCC patients

	PFS			OS		
	HR	95% CI	*P* value	HR	95% CI	*P* value
Age	0.981	0.942–1.02	0.368	0.923	0.842–1.012	0.089
Sex	1.206	0.636–2.28	0.565	1.306	0.369–4.624	0.679
CTP score	0.944	0.598–1.49	0.806	1.814	0.767–4.287	0.175
MELD score	0.980	0.855–1.12	0.775	1.118	0.877–1.426	0.367
ECOG PS	1.528	0.691–3.380	0.296	3.050	0.610–15.261	0.175
Metastasis	0.64	0.363–1.129	0.123	1.203	0.318–4.547	0.785
HCV	2.028	0.744–5.533	0.167	5.603	0.732–42.868	0.097
Diabetes mellitus	1.011	0.469–2.181	0.977	1.795	0.272–11.827	0.543
Hypertension	0.996	0.458–2.168	0.993	0.991	0.123–7.945	0.993
Sorafenib average dose	0.994	0.991–0.997	0.0001*	0.983	0.975–0.991	0.0001*
IL-23R rs7517847						
TT (reference)						
GT	Reference	Reference	0.097	Reference	Reference	0.043*
GG	2.095	1.067–4.116	0.032*	3.283	0.839–12.843	0.088
	2.003	0.613–6.542	0.250	11.595	1.664–80.794	0.013*
ATG-10 rs10514231						
CC (reference)						
CT	Reference	Reference	0.028*	Reference	Reference	0.009*
TT	0.699	0.359–1.361	0.293	0.1	0.021–0.484	0.004*
	0.289	0.117–0.717	0.007*	0.093	0.008–1.036	0.053

Values in bold with an asterisk (*) represent statistically significant results (*P* ≤ 0.05)

CTP, Child–Turcotte–Pugh; ECOG, Eastern Cooperative Oncology Group; MELD, model for end-stage liver disease; HCV, hepatitis C virus; OS, overall survival; PFS, progression-free survival; and CI, confidence interval

On the contrary, the ATG-10 rs10514231 polymorphism was significantly associated with both OS and PFS. Patients carrying the TT genotype exhibited a prolonged OS (mean survival time: 383 days, 95% CI 301.1–354.3), compared to CC carriers (289.5 days, 95% CI 228.5–350.1, *P* = 0.025). Additionally, TT carriers had a longer PFS (311 days, 95% CI 199.4–247.9) than CC carriers (207.7 days, 95% CI 163.4–252.1, *P* = 0.011), indicating a favorable prognosis. Cox regression analysis further confirmed that the TT genotype was associated with a significantly lower risk of disease progression (HR = 0.289, *P* = 0.007) and mortality (HR = 0.293, *P* = 0.004), reinforcing its potential protective effect. Other clinical factors, including age, sex, CTP score, MELD score, presence of metastases, diabetes mellitus, hypertension, and HCV infection, did not exhibit statistically significant associations with survival outcomes except the average dose of Sorafenib that was significantly associated with both PFS (HR = 0.994, 95% CI = 0.991–0.997, *P* < 0.0001) and OS (HR = 0.983, 95% CI = 0.975–0.991, *P* < 0.0001), indicating that higher Sorafenib doses were correlated with improved survival outcomes.

### Genotypes and average Sorafenib dose

As shown in Table 7, a one-way ANOVA was performed to examine the effect of IL-23R (rs7517847) and ATG-10 (rs10514231) genotypes on average Sorafenib dosage. The analysis revealed a significant difference in Sorafenib dosage across IL-23R genotypes (*F* = 4.14, *P* = 0.019), whereas no significant difference was observed for ATG-10 (*F* = 2.52, *P* = 0.086). Post hoc Tukey HSD analysis confirmed that patients carrying the GG genotype received significantly higher doses compared to TT (*P* = 0.0174) and GT (*P* = 0.0227), while TT and GT groups did not differ significantly (*P* = 0.9856).

**Table 7:** Genotypes and average Sorafenib dose

Parameter	IL-23R (rs7517847)				
	TT (mean ± SD)	GT (mean ± SD)	GG (mean ± SD)	*F* value	*P* value
	(95%CI)	(95%CI)	(95%CI)		
Average dose	391.1 ± 105.63	394.85 ± 11.6	498 ± 119.04	4.139	0.019*
	(359.36–422.84)	(361.3–428.4)	(412.8–583.16)		

Parameter	ATG-10 (rs10514231)				
	CC (mean ± SD)	CT (mean ± SD)	TT (mean ± SD)	*F* value	*P* value
	(95%CI)	(95%CI)	(95%CI)		
Average dose	423.7 ± 123.5	379.2 ± 108.4	434.7 ± 102.2	2.517	0.086
	(374.8–472.6)	(348.77–409.77)	(381–425.9)		

Results are reported as mean ± standard deviation (95% confidence interval)

Values in bold with an asterisk (*) represent statistically significant results (*P* ≤ 0.05)

### Sorafenib-related adverse events

Table 8 outlines the adverse events related to drug administration that were documented during the study. Among the 100 patients who received Sorafenib, the most reported adverse event was fatigue (84%), followed by increased blood pressure (65%), diarrhea (41%), and Hand–Foot Syndrome Reaction (HFSR) (38%). The severity of adverse events was classified according to the National Cancer Institute Common Terminology Criteria. The statistical evaluation indicated no substantial correlation between the majority of adverse events and the examined polymorphisms (*P* > 0.05). However, HFSR Grade 2 showed a significant correlation with the ATG-10 genotype (*P* = 0.012), suggesting a potential genetic predisposition in patients carrying certain CC, CT, or TT variants. An ANCOVA analysis was performed to examine the association between the ATG-10 genotype and Hand-Foot Skin Reaction (HFSR), adjusting for Sorafenib dosage. Notably, higher Sorafenib doses were independently linked to increased HFSR severity (*F* = 4.90, *P* = 0.0292). Even after controlling the Sorafenib dosage, the ATG-10 genotype remained a significant predictor of HFSR severity (*F* = 6.73, *P* = 0.0018). Other adverse effects, including increased blood pressure, diarrhea, renal toxicity, and anorexia, did not exhibit statistically significant differences across genotypic variations.

**Table 8:** Sorafenib recorded adverse events with IL-23R and ATG-10 genotype

Adverse effect		Total number *n* = 100	IL-23R			*X*^2^	*P*	ATG-10			*X*^2^	*P*
			rs7517847 *n* total = 100					rs10514231 *n* total = 100				
			TT	GT	GG			CC	CT	TT		
			N	N	N			N	N	N		
Increase blood pressure	Grade 1	53	26	18	9	10.10	0.12	13	26	14	2.83	0.830
	Grade 2	9	3	5	1			2	6	1		
	Grade 3	3	1	2	0			1	2	0		
Bleeding adverse event	Grade 1	12	5	7	0	8.85	0.182	3	7	2	6.3	0.386
	Grade 2	4	0	4	0			3	1	0		
	Grade 3	1	0	1	0			0	1	0		
Diarrhea	Grade 1	33	13	14	6	9.861	0.131	9	18	6	5.107	0.530
	Grade 2	5	4	1	0			1	2	2		
	Grade 3	3	3	0	0			0	1	2		
Constipation	Grade 1	3	2	1	0	1.99	0.737	1	1	1	1.365	.850
	Grade 2	1	1	0	0			0	1	0		
Renal toxicity	Grade 1	6	4	2	0	1.497	0.473	0	3	3	3.99	0.135
HFSR	Grade 1	27	8	16	3	5.635	0.252	6	13	8	12.794	0.012*
	Grade 2	11	7	4	0			0	5	6		
Fatigue	Grade 1	58	28	24	6	1.767	0.940	15	29	14	1.805	0.937
	Grade 2	23	9	11	3			7	11	5		
	Grade 3	3	1	2	0			1	1	1		
Anorexia	Grade 1	10	3	4	3	5.062	0.08	0	6	4	4.813	0.09
Headache	Grade 1	6	2	4	0	1.497	0.473	2	2	2	0.858	0.651
Itchiness	Grade 1	6	5	1	0	4.395	0.355	1	1	4	8.613	0.072
	Grade 2	4	2	2	0			1	3	0		
Hepatic encephalopathy	Grade 1	1	0	1	0	3.78	0.437	0	0	1	4.390	0.356
	Grade 2	2	0	2	0			1	1	0		
Abdominal pain	Grade 1	12	3	8	1	4.933	0.552	4	4	4	5.491	0.483
	Grade 2	1	1	0	0			0	1	0		
	Grade 3	1	1	0	0			1	0	0		
Ascites	Grade 2	7	4	3	0	1.007	0.604	2	3	2	0.252	0.881
Bone ache	Grade 1	9	4	5	0	3.731	0.713	2	4	3	2.659	0.850
	Grade 2	1	1	0	0			0	1	0		
	Grade 3	1	0	1	0			0	1	0		

The severity of adverse events was evaluated according to the National Cancer Institute Common Terminology Criteria for Adverse Events, version 5.0

Values in bold with an asterisk (*) represent statistically significant results (*P* ≤ 0.05)

## Discussion

Sorafenib remains one of the standard therapies for advanced HCC [31]. Yet, Sorafenib resistance is very common, and many patients show treatment progression within 6 months [14]. The availability of alternative treatments has led to the need for informative biomarkers to predict Sorafenib response [32]. These biomarkers optimize patient care by minimizing treatment burden and avoiding adverse effects [12]. The human genome contains more than 14 million polymorphisms, and it has been known that SNPs can affect drug response variability [33]. To the best of our knowledge, this is the first clinical study exploring the potential prognostic significance of IL-23R rs7517847 and ATG-10 rs10514231 in predicting Sorafenib response in advanced HCC patients.

As a key component of immune regulation, IL-23R significantly influences innate immunity and inflammatory signaling, influencing the development, progression, and therapeutic response of many cancers including HCC [17, 19, 21, 22]. Based on our findings, the IL-23R genotype significantly impacted patient outcomes. Patients with the TT genotype had a higher partial response rate (PR) and overall response (OAR) of 55.9% compared to GT and GG genotypes. These results suggest that IL-23R polymorphisms may contribute to differences in immune response, potentially influencing tumor aggressiveness and drug sensitivity [22]. Previous research on Egyptian patients with HCC revealed that the IL-23R rs7517847 G allele and GG genotype were significantly more frequent in HCC patients than in healthy controls [17]. This aligns with our findings that GG carriers exhibited a poorer response to Sorafenib. Moreover, IL-23R is generally expressed in activated macrophages, memory T cells, monocytes, and dendritic cells which play an important role in immunity response [34]. Additionally, IL-22, which is functionally associated with IL-23R signaling, has been implicated in promoting Sorafenib resistance in HCC through STAT3 activation, leading to enhanced tumor cell survival and decreased apoptosis. This signaling cascade further facilitates immune evasion by impairing natural killer cell effectiveness [35]. High expression of tumor-associated macrophages and cancer stem cell markers contributes to an immunosuppressive tumor microenvironment, potentially intersecting with IL-23/IL-23R signaling [36].

Research findings suggest that IL-23R plays a pivotal role in inflammatory diseases, including ankylosing spondylitis (AS), inflammatory bowel disease (IBD), Crohn’s disease (CD), psoriasis, and multiple sclerosis, with evidence supporting the protective effects of certain IL-23R gene variants, which strongly supports our hypothesis (Jezernik et al., 2023; Mm, 2012; Zhang et al., 2015). On the contrary, no correlation was identified between the IL-23R gene rs7517847 T > G SNP and systemic lupus erythematosus (SLE) or ulcerative colitis (UC) [37, 38]. This may be attributed to the autoinflammatory response of the innate immune system in AS, in contrast with autoimmune disorders such as rheumatoid arthritis (RA), which engage the adaptive immune system [39, 40].

While Kaplan–Meier analysis did not show significant survival differences, Cox regression identified the IL-23R GG genotype as a significant predictor of poor OS. This discrepancy likely due to the lower representation of participants in the GG group (*N* = 10) compared to TT (*N* = 45) and GT (*N* = 45). Our study reported that IL-23R polymorphisms influence the average tolerable Sorafenib dose, with GG carriers receiving significantly higher doses compared to TT and GT genotypes. This suggests that IL-23R variants may modulate drug metabolism or influence treatment tolerability, necessitating dose adjustments for certain genotypes. The biological mechanism underlying this association remains unclear, but it is possible that IL-23R-mediated immune responses and inflammation indirectly affect hepatic drug metabolism and clearance, altering Sorafenib pharmacokinetics [16, 41–43]. Interestingly, despite its impact on dosing, IL-23R polymorphisms were not significantly correlated with any adverse events, suggesting that IL-23R may not contribute to Sorafenib-induced toxicity. Inflammation in hepatocellular carcinoma (HCC) is a key factor in treatment resistance and understanding IL-23R's impact could provide insights into patient variability in drug response, potentially improving Sorafenib efficacy and enhancing treatment outcomes [16].

Moreover, our results demonstrate that genetic variations in ATG-10 markedly affected treatment outcomes, reinforcing its role in autophagy regulation and cancer therapy resistance. Notably, the TT genotype was associated with superior treatment response, extended PFS, and improved OS compared to the CT and CC genotypes, supporting ATG-10’s function in modulating Sorafenib efficacy. Autophagy is a cellular process that recycles and degrades damaged components, which ATG genes play a vital role in regulating it, is linked to cancer progression, including resistance to chemotherapy and targeted therapies like Sorafenib [44].

Recent research has shown that autophagy can either promote or inhibit cancer progression, depending on the context. In some cases, autophagy facilitates cancer cell survival under stress, such as chemotherapy, via inhibiting the accumulation of defective proteins and organelles [45]. This aligns with our findings that patients possessing the TT genotype, linked to enhanced autophagic activity, exhibited superior clinical results. Excessive autophagy was found to be a promoter for apoptosis in tumor cells when pemetrexed (autophagy stimulator) was added to Sorafenib in vivo experiment and enhanced the Sorafenib response [26]. This suggests that the precise regulation of autophagy played an essential function in determining the effectiveness of Sorafenib, as excessive autophagy could sensitize tumor cells to treatment. However, this finding contrasts with some studies that suggest autophagy contributes to drug resistance in HCC and other cancers [46, 47]. The discrepancy between studies may be ascribed to variations in the experimental models used, the type of autophagy inducer, or the genetic background of the tumor cells. Therefore, additional studies are required to clarify the complex role of autophagy in cancer treatment, particularly in the context of Sorafenib resistance. Additionally, our findings align with the previous studies suggesting that ATG-10 polymorphisms may influence cancer prognosis. Specifically, the C allele is associated with enhanced luciferase activity and increased gene expression in both HCC and normal hepatic tissues, highlighting ATG-10 rs10514231 as a potential prognostic marker for liver cancer [27]. The study we conducted expands upon existing findings by illustrating that ATG-10 influences cancer growth and is crucial in forecasting therapeutic outcomes for patients undergoing Sorafenib treatment.

Also, epidemiological findings suggest that ATG gene variants may modulate biological activity, thereby affecting cancer susceptibility and clinical outcomes in malignancies such as lung cancer and HCC [48]. Moreover, a study on 468 nasopharyngeal carcinoma (NPC) patients found that ATG-10 rs10514231 significantly reduced radiation efficacy in the primary tumor and lymph node, indicating a potential link between functional ATG and radiation therapy effectiveness [49]. Furthermore, consistent with our findings, a previous study reported that ATG-10 can be used as a promising clinical predictor in advanced lung adenocarcinoma patients for gefitinib response which is an EGFR-TKI similar to Sorafenib [25]. Furthermore, our results indicate that ATG-10 CT carriers had a significantly higher risk of developing Hand–Foot Syndrome, independent of Sorafenib dose (ANCOVA, *P* = 0.0018). These findings suggest a genetic predisposition to toxicity, reinforcing the need for genotype-guided dosing strategies to mitigate adverse events.

Additionally, in our study, the average tolerable dose of Sorafenib was 403.4 ± 113.1 mg/day, which is notably lower than the standard recommended dose of 800 mg/day [43]. This finding aligns with the previous research suggesting that many HCC patients struggle to tolerate the full dose due to severe adverse events, necessitating dose reductions to maintain treatment adherence [50]. Dose modification strategies have been shown to improve patient survival by minimizing adverse effects while maintaining drug efficacy [51]. In real-world clinical practice, an initial dose reduction to 400 mg/day, rather than maintaining 800 mg/day, has been associated with better treatment adherence, lower incidence of dose-related toxicities, and longer progression-free survival [50].

In conclusion, our study identifies IL-23R and ATG-10 as potential biomarkers for Sorafenib response, survival outcomes, and toxicity risk in HCC patients. IL-23R polymorphisms impact both drug response and dosage adjustments, whereas ATG-10 influences survival and adverse event susceptibility. These findings emphasize the critical role of inflammation and autophagy in Sorafenib efficacy, suggesting that genotype-based treatment strategies could optimize therapeutic outcomes. Future research should explore personalized dosing algorithms to enhance efficacy and minimize toxicity in advanced HCC patients.

## Conclusion

Sorafenib is preserved for advanced-stage HCC patients who are not eligible for resection and other therapies. This makes the time factor even more important in the management of patients eligible for Sorafenib, particularly with the emergence of novel therapies. The availability of genetic markers for early detection of Sorafenib resistance which can help in avoiding unnecessary expenditure of patient’s time and resources and in maximizing the benefit from alternative treatments would be super beneficial. Both ATG-10 and IL-23R polymorphism investigated in this study showed promising findings to act as Sorafenib response predictors with even a lower cost than 1 week of therapy and without experiencing undesirable side effects.

This research offers new perspectives on the role of IL-23R and ATG-10 polymorphisms in Sorafenib response, survival outcomes, and tolerability in HCC patients, but certain limitations should be considered. The constrained number of enrolled patients (*N* = 100), particularly the low representation of the IL-23R GG genotype (*N* = 10), may have limited statistical power. Additionally, the single-center design and the 6-month follow-up may not fully capture long-term survival outcomes and late-onset toxicities. While genetic associations were established, functional analyses were not performed to explore the underlying molecular mechanisms. Future research should focus on multi-center validation, functional studies, and genotype-guided Sorafenib dosing models to enhance treatment personalization and optimize patient outcomes.

## Supplementary Information

Below is the link to the electronic supplementary material.

Supplementary file1 (XLSX 20 KB)

## Data Availability

The data that support the findings of this study are available from the corresponding author upon request.
